# A 2022 Update on Computational Approaches to the Discovery and Design of Antimicrobial Peptides

**DOI:** 10.3390/antibiotics12061011

**Published:** 2023-06-05

**Authors:** Guillermin Agüero-Chapin, Agostinho Antunes, Yovani Marrero-Ponce

**Affiliations:** 1 CIIMAR/CIMAR, Interdisciplinary Centre of Marine and Environmental Research, University of Porto, 4450-208 Porto, Portugal; 2 Department of Biology, Faculty of Sciences, University of Porto, 4169-007 Porto, Portugal; 3 Universidad San Francisco de Quito (USFQ), Grupo de Medicina Molecular y Traslacional (MeM&T), Colegio de Ciencias de la Salud (COCSA), Escuela de Medicina, Edificio de Especialidades Médicas, Diego de Robles y vía Interoceánica, Quito 170157, Pichincha, Ecuador; 4 Departamento de Ciencias de la Computación, Centro de Investigación Científica y de Educación Superior de Ensenada (CICESE), Ensenada 22860, Baja California, Mexico

The antimicrobial resistance process has been accelerated by the over-prescription and misuse of antibiotics. The World Health Organization (WHO) has listed it as one of the top 10 global public health threats. This worrisome situation has encouraged the search for new classes of antimicrobial agents, leveraging the ability of antimicrobial peptides (AMPs) to overcome resistance, mainly due to their versatile mode of action and multifunctionalities. However, the discovery of promising AMPs with relevant biological activities is a real challenge, considering the great structural diversity of the AMP class and their under-representation in terms of non-bioactive peptides. Consequently, several databases and computational approaches have been developed for over two decades to assist in the long development process of peptide-based drugs.

This Special Issue, entitled “*Computational Approaches to the Discovery & Design of Antimicrobial Peptides*,” is mainly dedicated to state-of-the-art in silico approaches applied to the discovery and design of AMPs for therapeutic purposes. In this sense, Agüero-Chapin et al. published a comprehensive review article on emerging in silico approaches to the search for/design of bioactive peptides, from new machine learning (ML) algorithms to other non-conventional methodologies, such as complex networks and algorithms simulating peptide sequence evolution. New considerations incorporated into the biodiscovery workflow for unravelling AMPs from omics data were also analyzed [1].

Aligning with the previously mentioned review, Ruiz-Blanco et al. developed a new machine learning (ML)-based classifier for the detection of antibacterial peptides (ABPs) and their putative targets, including multi-drug-resistant (MDR) bacterial strains. The ML model was implemented in a web server called “ABP-Finder”, which is one of the most state-of-the-art ABP predictors, with a proven high precision when detecting a promising peptide hit against *P. aeruginosa* during the screening of large databases such as the human urine peptidome [2]. The revision also comprised non-conventional methodologies applied to the field of AMPs. For example, García-Hernández et al. repurposed ROSE (Random Model of Sequence Evolution), an algorithm simulating sequence evolution, to generate diversity-oriented libraries of peptides as one of the steps for the de novo design/optimization of antibacterial peptides (ABPs) by inhibiting the E. coli FoF1-ATP synthase [3]. 

On the other hand, Marrero-Ponce et al. applied network science to study the chemical space of tumor-homing peptides (THPs) by using alignment-free similarity networks and centrality measures to identify the most relevant and non-redundant THPs within the network. Such THPs, representing the original TH chemical space, were considered as references for multi-query similarity searches that apply a group fusion (MAX-SIM rule) model. The resulting multi-query similarity searching models outperformed state-of-the-art predictors in the detection of THPs in benchmark datasets. This approach also served to search for THP leads and to discover TH motifs [4].

Related to the previously discussed on the new considerations and tools incorporated into the workflow for AMP biodiscovery [1], Birol et al. developed rAMPage, a scalable bioinformatics tool for identifying AMP sequences from RNA sequencing (RNA-seq) datasets. rAMPage was extensively evaluated on publicly available RNA-seq datasets from amphibian and insect species. It identified 1137 putative AMPs, of which 1024 were considered novel by homology criteria. From these, 21 peptides were tested for antimicrobial susceptibility against two bacteria species, *E. coli* and *S. aureus*, and 7 showed high activity. Thus, rAMPage can be integrated into the workflow for AMP biodiscovery to accelerate the process of antimicrobial drug development [5]. 

Although transcriptomic and proteomic analyses can streamline the biodiscovery workflow of AMPs by focusing on gene coding and protein expression, such high-throughput screening can be performed at the genomic level to unravel both encoded and cryptic AMPs. Hancock et al. proposed profile hidden Markov models to screen the genomes of four crocodilian species for identifying encoded cathelicidin sequences. Cathelicidins are one of the largest family of host defense peptides, showing a broad-spectrum activity against planktonic bacteria and some biofilm, as well as other beneficial features such as anti-inflammatory properties. Eighteen novel cathelicidin sequences were identified and subsequently synthetized and evaluated in vitro against planktonic and biofilm bacteria. Among the cathelicidins which displayed a broad-spectrum antimicrobial and antibiofilm activity against a range of antibiotic-resistant bacteria, As-CATH8 was highlighted because of its similar profile to the last-resort antibiotics vancomycin and polymyxin B [6]. An alternative method of searching for AMPs at the genomic level involves the in silico detection of corresponding biosynthetic gene clusters (BGCs). Ashraf et al. sequenced the genome of the Streptomyces sp. isolate BR123 and used the online antiSMASH (antibiotics & Secondary Metabolite Analysis Shell) platform to analyze the resulting assembled regions. Multiple BGCs were detected that were involved in the production of antimicrobial, antiparasitic, and anticancer compounds [7]. 

Two additional review papers were published in this Special Issue. Rivera-Fernández et al. examined the experimental effects of various bioactive peptides on Apicomplexan parasites, which are responsible for a range of dangerous diseases, such as toxoplasmosis, cryptosporidiosis, and malaria. They also discussed some biological and metabolomic generalities of the parasites to explain the mechanisms of action of the peptides on the Apicomplexan targets [8]. The other review paper was written by Prof. Juretić, which emphasizes the importance of designing multi-functional peptides that can reach intracellular targets in order to develop more effective peptide drugs. The review ranked known and novel peptides based on their predicted low toxicity to mammalian cells and broad-spectrum activity. The 20 most promising candidates that exhibited optimized cell-penetrating, antimicrobial, anticancer, anti-viral, antifungal, and anti-inflammatory activities were identified. These peptides also have the ability to form an amphipathic structure upon contact with membranes or nucleic acids [9]. 

Prof. Juretić’s work also mentioned the urgent need to develop antifungal compounds that target intracellular molecules as a strategy to combat multidrug-resistant (MDR) pathogens such as *Cryptococcus neoformans*, which pose a threat to immunocompromised patients. Consequently, the study by Souza et al. designed and tested anticryptococcal AMPs and provided further information on their mechanism of action against *C. neoformans* using computational and experimental analyses [10].
